# Homœopathy and the Michigan University

**Published:** 1876-04

**Authors:** 


### Homœopathy and the Michigan University.

The Regents of the University of Michigan, in establishing Chairs of Homœopathy in that institution, seem to have aroused more of an opposition than they counted on.

To all disinterested parties, the establishment of a Homœopathic College with two professors professing that dogma, the balance of the teaching being done by the regular faculty, makes the old faculty part of the new one also. There seems to us to be but very little difference between the man who teaches anatomy, surgery or obstetrics to the Homœopathic students, and examines and passes them as such, *but does not sign their diplomas*, and the man who does all this in a Homœopathic school, and *does sign the diplomas* as a Homœopathic professor.

The propriety of the State teaching medicine we have always doubted; the manifest impropriety of it the University of Michigan is at present demonstrating to the world. It seems to us no more proper that the State of Michigan, or any other State, should establish a school for instruction in any one theory of medicine than that it should teach one form of religion to the exclusion of all others. If it teaches Presbyterianism or Methodism, the people have a right to demand that it shall teach Catholicism or Spiritualism. So with medicine; the State has no more right to tax its subjects to support a school of regular medicine than it has to support one of Homœopathy, Eclecticism, or any other medical vagary that may spring up.

But there is another side to the question. While the profession cannot demand that the State shall teach regular medicinc and nothing else, they can demand that those who have hitherto been honored and respected as exponents of true, scientific medicine, shall not digrace themselves and the profession to which they belong by lending themselves as assistants to a Homœopathic Faculty in teaching the very branches which Homœopathic professors would otherwise teach. While the Medical Faculty of the University of Michigan are thus under a cloud, the profession can do nothing less than refuse to place pupils under their instruction, and we believe the question is already under discussion whether medical colleges can recognize certificates of attendance upon lectures issued by this Faculty.

We append a letter addressed to the Alumni Association of the Medical Department, University of Michigan, by a number of graduates residing in Toledo, Ohio. The letter plainly exhibits the feeling of the profession upon this subject:

TOLEDO, O., March 23, 1876.

*W. F. Breaky, M. D., Secretary Alumni Association, Medical Department, University of Michigan :*

DEAR SIR—Believing that a duty is incumbent upon those who have already graduated in the Medical Department of the University, we desire, through you, to express to her Alumni Association our views in regard to the introduction of Homœpathy into that institution.

We, in common with many others, have always referred with pride to the diploma received from the regents of the University, and with corresponding pleasure to the "Honored Faculty" that in those days wrote thereon their names and titles. This evidence of our professional ability we held to be "Armor and Shield." We have watched with eagerness the development of the Medical Department of the University, and her immense power for good in the profession. Her honors were measureably our own, and having shared her glory, in an evil day we must likewise bear a portion of her shame. Our hearts have been bound in bonds of love to her, as well as in duty to the profession to which her gift wedded us. We could, in the fullness of our hearts, forgive much in our *Alma Mater*, but the principles of our profession dictate that love shall never interfere with duty. Our relation to this question is totally non-partisan, and void of all personal feeling. We are free from the local influences that bear upon many others, and care nothing for the adroit logic and language with which it is attempted to cover the startling fact, that a pretended system of medicine, a system which we were then taught to believe, and now conscientiously hold to be, the most arrant attempt at humbuggery, is engrafted upon the institution.

We shudder at the fact that a Faculty in part composed of those whose names appear upon our diplomas, now join hands with those who teach this unblushing charlatanism, by freely giving their students the benefit of additional instruction, thus becomiug an organized corps of co-laborers in preparing them to practice a pretended system that not only shames science, but is a byword to simple common sense. When the advocates of *simila similibus curantur*, and the law that the less the dose of a given drug the more powerful it proves to be, flaunt in our faces their diplomas, bearing the same name and seal as our own, we can but estimate the value of ours, obtained when her name was without stain, as very sadly depreciated. Words of reproach we have none, and our sentiments are only those of sorrow. Our *Alma Mater* is smitten with a loathsome disease. We hope that its fatality may be averted, and to this end respectfully express our belief and feelings that the alliance should not be continued, that at all hazards the purity of medical teachings should there be maintained ; that, come the very worst, we enter our earnest and solemn protest against the teaching of Homœ-

opathy, or even its tacit recognition in the Medical Department of the University of Michigan. Very respectfully, &c.,

[Signed],
J. T. WOODS, M. D., Class of 1855.
GEO. W. BOWEN, M. D., Class of 1860
J. F. ARIS, M. D., Class of 1865,
JAS. A. DUNCAN, M. D., Class of 1871.
EEWARD M. HALL, M. D., Class of 1873.
JAS. T. LAWLESS, M· D., Class of 1872.
A. J. BOSTATER, M. D., Class of 1871.
C. R. HUME, M. D., Class of 1874.

